# Kinetics of recruitment and allosteric activation of ARHGEF25 isoforms by the heterotrimeric G-protein Gαq

**DOI:** 10.1038/srep36825

**Published:** 2016-11-11

**Authors:** Jakobus van Unen, Taofei Yin, Yi I. Wu, Marieke Mastop, Theodorus W. J. Gadella, Joachim Goedhart

**Affiliations:** 1 Swammerdam Institute for Life Sciences, Section of Molecular Cytology, van Leeuwenhoek Centre for Advanced Microscopy, University of Amsterdam, P.O. Box 94215, NL-1090 GE Amsterdam, The Netherlands; 2 Center for Cell Analysis and Modeling, University of Connecticut Health Center, 400 Farmington Avenue, Farmington, CT 06032-6406, USA.

## Abstract

Rho GTPases are master regulators of the eukaryotic cytoskeleton. The activation of Rho GTPases is governed by Rho guanine nucleotide exchange factors (GEFs). Three RhoGEF isoforms are produced by the gene ARHGEF25; p63RhoGEF^580^, GEFT and a recently discovered longer isoform of 619 amino acids (p63RhoGEF^619^). The subcellular distribution of p63RhoGEF^580^ and p63RhoGEF^619^ is strikingly different in unstimulated cells, p63RhoGEF^580^ is located at the plasma membrane and p63RhoGEF^619^ is confined to the cytoplasm. Interestingly, we find that both P63RhoGEF^580^ and p63RhoGEF^619^ activate RhoGTPases to a similar extent after stimulation of Gαq coupled GPCRs. Furthermore, we show that p63RhoGEF^619^ relocates to the plasma membrane upon activation of Gαq coupled GPCRs, resembling the well-known activation mechanism of RhoGEFs activated by Gα_12/13_. Synthetic recruitment of p63RhoGEF^619^ to the plasma membrane increases RhoGEF activity towards RhoA, but full activation requires allosteric activation via Gαq. Together, these findings reveal a dual role for Gαq in RhoGEF activation, as it both recruits and allosterically activates cytosolic ARHGEF25 isoforms.

Rho GTPases are best known for their regulation of the cytoskeleton in eukaryotic cells^1^. They function as molecular switches that transition between an active GTP-bound form and an inactive GDP-bound form^2^. Rho guanine exchange factors (RhoGEFs) accelerate the exchange of GDP for GTP on RhoGTPases^3^, whereas Rho GTPase activating proteins (RhoGAPs) catalyze the hydrolysis of GTP to GDP on the Rho GTPase^4^.

The link between G-protein coupled receptors (GPCRs) and Rho GTPase signaling is well established^5^,^6^,^7^,^8^. It was initially shown that heterotrimeric G-proteins of the Gα_12/13_ family are involved in the activation of RGS containing RhoGEFs for the RhoA family of Rho GTPases^9^. More recently, the role of G-proteins of the Gαq family in GPCR mediated activation of RhoA was discovered^10^,^11^. Trio^12^ and p63RhoGEF^13^ are RhoGEFs that are activated by direct interaction with Gαq. Biochemical and structural studies have well established that activation of the heterotrimeric G-protein Gαq relieves the DH domain of p63RhoGEF from its auto-inhibited state by allosteric interaction with the PH domain^12^,^14^,^15^.

P63RhoGEF (encoded by the gene ARHGEF25) mediates activation of RhoA by Gαq in smooth muscle cells^16^,^17^ and has been proposed as a key regulator of angiotensin II induced effects on vascular smooth muscle tissue^18^,^19^. The ARHGEF25 gene encodes for several isoforms, which are indicated as GEFT or p63RhoGEF. It is not always clear which isoform is used in a particular study. Here we use p63RhoGEF^580^ for the 580 a.a. protein with a predicted mass of 63 kDa. The shorter isoform lacking 106 a.a. of the N-terminus is termed GEFT here. Both p63RhoGEF^580^ and GEFT specifically activate RhoGTPases of the RhoA family *in vitro*^12^,^20^ and in cultured cells^21^. Despite their similar functionality towards RhoA, p63RhoGEF^580^ and GEFT have a remarkable different localization pattern inside cells. Palmitoylation of several cysteines at its N-terminus causes p63RhoGEF^580^ to be constitutively targeted to the plasma membrane in unstimulated cells, which is a unique feature for RhoA activating RhoGEFs. GEFT resides in the cytoplasm, it does not contain the first 106 a.a. of p63RhoGEF^580^, and thus lacks the N-terminal palmitoylation sites. The plasma membrane localization of both these p63RhoGEF isoforms have been shown to be important for their interaction with Gαq^22^,^23^.

More recently a third, longer variant encoded by ARHGEF25 was described in the literature^24^. This isoform has a different N-terminus, comprises 619 a.a. in total and is indicated here as p63RhoGEF^619^. It was found in several mass spectrometry based proteomic analyses of human tissues^25^,^26^. The p63RhoGEF^619^ variant is transcribed from two alternative N-terminal exons and, similar to GEFT, does not contain the N-terminal palmitoylation sites. The subcellular localization and functional properties of p63RhoGEF^619^ in relation to the other two isoforms have not yet been investigated. Furthermore, the mechanism of activation of ARHGEF25 isoforms in the cytoplasm by Gαq has not yet been elucidated.

Here we use advanced microscopy techniques to characterize the mechanism with which the different ARHGEF25 isoforms (p63RhoGEF^580^, GEFT and p63RhoGEF^619^) are activated by Gαq coupled GPCR stimulation. We show that although the subcellular localization of the different ARHGEF25 isoforms is strikingly different, they do not show any difference on basal and stimulated GEF activity towards RhoA. P63RhoGEF^580^ and p63RhoGEF^619^ can activate RhoA and Cdc42 via stimulation of endogenous Gαq coupled histamine-1-receptors (H_1_R). Furthermore we show that p63RhoGEF^619^ relocates to the plasma membrane upon activation of Gαq. Using a chemical dimerization system, we are able to show that recruitment of p63RhoGEF^619^ to the plasma membrane increases RhoGEF activity towards RhoA, but full activation requires allosteric activation by Gαq. This study highlights the dual function of Gαq, recruitment and allosteric activation, in the activation of cytosolic ARHGEF25 isoforms.

## Results

### The longer isoform of ARHGEF25 is located in the cytoplasm

P63RhoGEF/GEFT was first identified as a transforming gene product in a retroviral screen for oncogenes^27^,^28^. Subsequently it was found that the ARHGEF25 locus produces two isoforms, p63RhoGEF^580^ (580 a.a.) and its shorter variant GEFT (474 a.a.)^20^. More recently, a third and longer variant was described^24^, p63RhoGEF^619^, which is transcribed from two alternative exons at the N-terminus, and contains 619 a.a. in humans (see Supplemental Fig. 1 for a full sequence alignment of the three isoforms).

**Figure 1:**
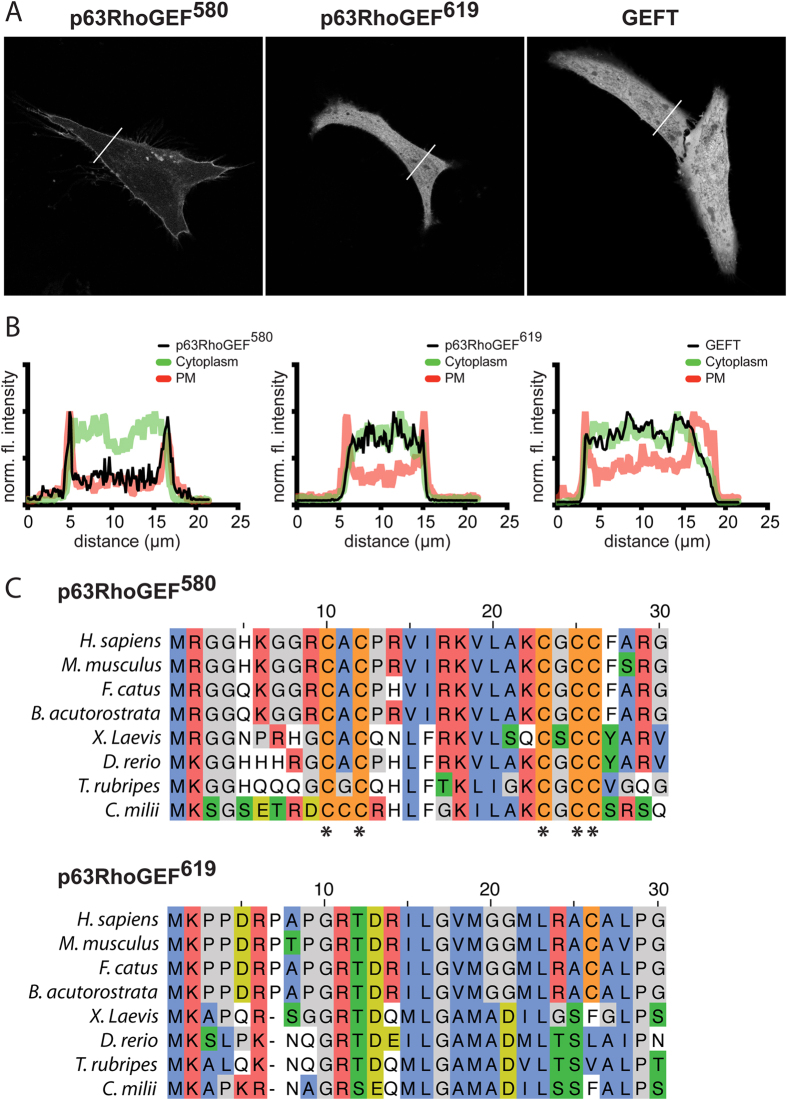
Localization and origin of ARHGEF25 isoforms. (**a**) Confocal images of HeLa cells expressing p63RhoGEF^580^-YFP (*left*), p63RhoGEF^619^-YFP (*middle*) or YFP-GEFT (*right*). (**b**) Line plots showing fluorescence intensities of the different GEF constructs along the lines indicated in (**a**), compared with the intensities of a co-transfected plasma membrane marker (Lck-CFP) and a cytosolic marker (RFP) transfected in the same cells. The intensities of the GEF, PM marker and cytosolic marker were normalized to the maximum fluorescent intensities in their respective channels. (**c**) Protein sequence homology analysis of the first N-terminal 30 amino acids of p63RhoGEF^580^ and p63RhoGEF^619^. Species compared: Human (H. sapiens), House mouse (M. musculus), Domestic cat (F. Catus), Mink whale (B. acutorostrata), African clawed frog (X. Laevis), Zebrafish (D. rerio), Pufferfish (T. rubripes) and Elephant shark/Australian ghost shark (C. milii). Amino acids are grouped by polar positive (*red*), polar negative (lightgreen), non-polar (blue), phosphorylation potential (green), palmitoylation potential (orange), or small (grey). Asterisks (*) mark the conserved potential palmitoylated cysteines of p63RhoGEF^580^. Width of the individual images in (**a**) is 121 μm.

To compare the subcellular localization of the different ARHGEF25 isoforms, HeLa cells were transfected with a plasma membrane (PM) marker (Lck-CFP), a cytosolic marker (RFP) and either p63RhoGEF^580^-YFP, p63RhoGEF^619^-YFP or YFP-GEFT. Confocal images of cells transfected with p63RhoGEF^580^-YFP show that p63RhoGEF^580^ is localized to the plasma membrane, as has been described before^22^,^23^. In contrast, cells transfected with either p63RhoGEF^619^-YFP or YFP-GEFT show only a cytosolic localization of these isoforms (Fig. 1A,B).

Previous studies have shown that p63RhoGEF^580^ is localized to the plasma membrane because of the palmitoylation of one or more cysteines at its the N-terminus^22^,^23^. In depth sequence analysis of the first 30 amino acids shows that the N-terminus, including cysteines, of p63RhoGEF^580^ is well conserved throughout the whole subphylum vertebrata (Fig. 1C, top). GEFT is essentially a splice variant of p63RhoGEF^580^ that does not contain the first 2 N-terminal exons of p63RhoGEF^580^ that contain the palmitoylated cysteines, resulting in cytoplasmic localization.

Sequence analysis of the relatively unexplored isoform p63RhoGEF^619^ shows that the N-terminus is well conserved among the vertebrate subphylum (Fig. 1C, bottom). There are no obvious PM targeting signals in the N-terminus, which is in agreement with the cytoplasmic localization observed in Fig. 1A. Of note, there seems to be a well-conserved ‘GRTD’ sequence at amino acid 10-13. Evolutionary sequence analysis (see Methods for details) reveals that both p63RhoGEF^580^ and p63RhoGEF^619^ are not found outside the vertebrate subphylum, and even so-called early or ancient vertebrate species like the *Lancelets*^29^ and *Lampreys*^30^ do not contain either isoform. Our results fit with a role for ARHGEF25 in smooth muscle (see discussion). To conclude, we find that p63RhoGEF^580^ is located at the plasma membrane, whereas the two other isoforms show cytoplasmic distribution.

### The basal GEF activity of ARHGEF25 isoforms is low in cells

**Figure 2:**
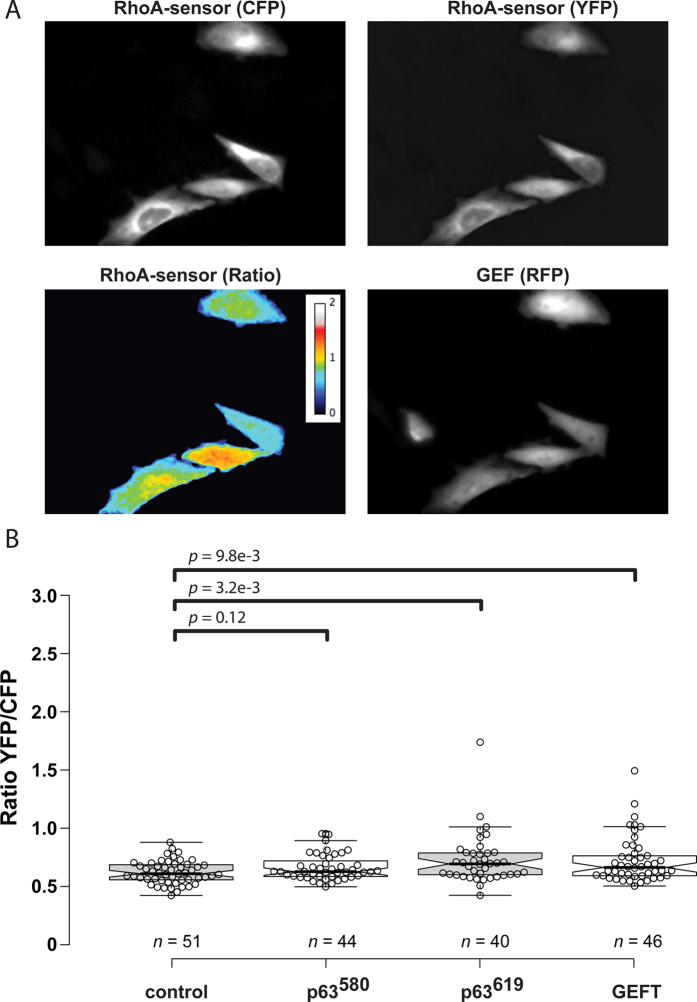
Basal RhoA activity of ARHGEF25 isoforms. (**a**) Example images of HeLa cells transfected with the DORA RhoA biosensor and p63RhoGEF^619^, showing the background corrected CFP, YFP, Ratio between YFP/CFP of the DORA RhoA biosensor and the RFP channels. (**b**) Boxplot showing the median basal YFP/CFP ratio of the DORA RhoA biosensor in HeLa cells transfected with the DORA RhoA biosensor and a cytosolic RFP (n = 51, control), p63RhoGEF^580^-RFP (n = 44), p63RhoGEF^619^-RFP (n = 40) or RFP-GEFT (n = 46). Individual data points are plotted as open circles. Experiments performed on 5 or more separate coverslips. Centerlines represent the median values and notches represent the 95% confidence interval for each median. The p-values were determined by a two-tailed Mann-Whitney test. Width of the individual images in (**a**) is 236 μm.

It has been well established that p63RhoGEF^580^ can be activated through G-protein coupled receptors (GPCRs), via direct interaction with the heterotrimeric G-protein subunit Gαq^12^,^14^,^22^,^31^. Activated p63RhoGEF^580^ catalyzes the exchange of GDP for GTP on Rho GTPases of the RhoA subfamily^12^. Even though it is located at the plasma membrane, p63RhoGEF^580^ has been confirmed to have low GEF activity towards RhoA *in vitro*^12^ and in cells^21^ due to the auto-inhibition by its PH domain. To compare the GEF activity towards RhoA of the different ARHGEF25 isoforms in unstimulated cells, we transfected HeLa cells with the previously characterized DORA RhoA biosensor^21^ and p63RhoGEF^580^-RFP, p63RhoGEF^619^-RFP, RFP-GEFT or only a cytosolic RFP (control) (Fig. 2A). The ratio between YFP and CFP fluorescence of the DORA RhoA biosensor, which confers the GTP loading state of RhoA, was determined for control (median = 0.61, 95% CI = 0.58–0.66) p63RhoGEF^580^ (median = 0.63, 95% CI = 0.61–0.67), p63RhoGEF^619^ (median = 0.69, 95% CI = 0.61–0.73) or GEFT (median = 0.66, 95% CI = 0.61–0.72) (Fig. 2A,B). The values for p63RhoGEF^580^, p63RhoGEF^619^ and GEFT are slightly elevated compared to the control condition, but are in line with low basal activity previously reported for p63RhoGEF^580^ 21. These results demonstrate that all different ARHGEF25 isoforms have low basal activity towards RhoA in unstimulated cells.

### Both p63RhoGEF^580^ and p63RhoGEF^619^ activate RhoA and Cdc42

In order to investigate whether the difference in location translates to a functional difference between the ARHGEF25 isoforms, we used DORA FRET biosensors for RhoA^21^, Rac1^32^ and Cdc42^33^ to measure GEF specificity and activity in stimulated cells. Hela cells were used in this study, because these cells express the endogenous histamine-1 receptor that couple to Gαq. In previous experiments, we have shown that in control conditions, i.e. cells transfected with mCherry^34^ or inactive variants of p63RhoGEF^21^, RhoA-GTP levels are not elevated upon stimulation with histamine. When p63RhoGEF^580^-RFP is ectopically expressed in Hela cells, a rapid activation of RhoA is observed after stimulation of the cells with histamine.

**Figure 3:**
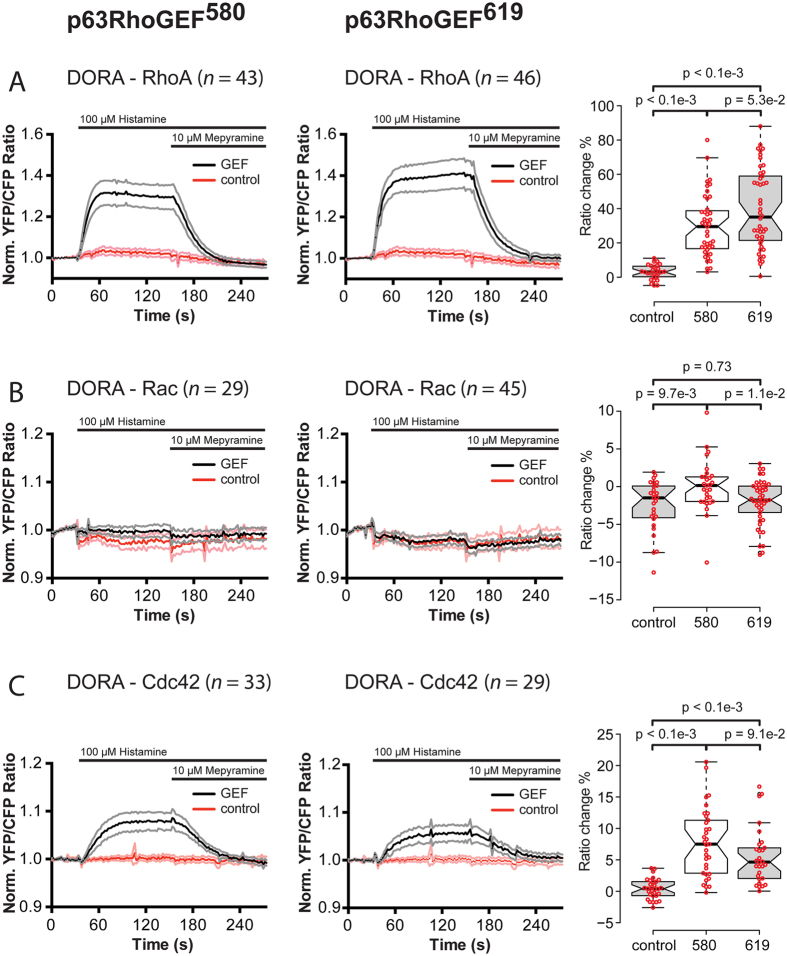
GPCR mediated activation of RhoA, Rac1 and Cdc42 by p63RhoGEF^580^ or p63RhoGEF^619^. (**a**) Time-lapse FRET ratio imaging of HeLa cells transfected with the DORA-RhoA biosensor and p63RhoGEF^580^ (*left, n* = 43) or p63RhoGEF^619^ (middle, n = 46) show a fast reversible increase in YFP/CFP ratio, indicating rapid GTP loading of RhoA upon GPCR stimulation. Control cells were transfected with the DORA-RhoA biosensor and a cytosolic RFP (*red traces, n* = 27). The median amplitude of the FRET ratio change at *t* = 120 seconds is shown on the *right*. (**b**) HeLa cells transfected with the DORA-Rac1 biosensor and p63RhoGEF^580^ *(left, n* = 29) or p63RhoGEF^619^ (*middle, n* = 45) show no changes in YFP/CFP ratio. Control cells were transfected with the DORA-Rac1 biosensor and a cytosolic RFP (*red traces, n* = 26). The median amplitude of the FRET ratio change at *t* = 120 seconds is shown on the *right*. (**c**) HeLa cells transfected with the DORA-Cdc42 biosensor and p63RhoGEF^580^ (*left, n* = 33) or p63RhoGEF^619^ (*middle, n* = 29) show a fast reversible increase in YFP/CFP ratio, indicating rapid GTP loading of Cdc42 upon GPCR stimulation. Control cells were transfected with the DORA-Cdc42 biosensor and a cytosolic RFP (*red traces, n* = 30). The median amplitude of the FRET ratio change at *t* = 120 seconds is shown on the *right*. Cells were stimulated with Histamine (100 μM) at t = 32 s and the response was antagonized by the addition of Mepyramine (10 μM) at t = 152 s. Time traces show the average ratio change of YFP/CFP fluorescence, normalized to baseline values (faded *grey* and *pink* traces depict +/−95% CI). Boxplots show individual data points and median values. Centerlines represent the median values and notches represent the 95% confidence interval for each median. The p-values were determined by a two-tailed Mann-Whitney test.

We transfected HeLa cells with p63RhoGEF^580^-RFP, p63RhoGEF^619^-RFP or a cytosolic RFP (control) and one of the three aforementioned Rho GTPase FRET sensors. CFP fluorescence and YFP sensitized emission were followed over time. Cells were stimulated with histamine to activate Gαq via endogenous histamine-1-receptors, and the response was antagonized with mepyramine. Stimulation of cells transfected with either p63RhoGEF^580^ or p63RhoGEF^619^ showed a reversible increase in YFP/CFP ratio of the DORA RhoA biosensor, with similar kinetic profiles (Fig. 3A). Stimulation of cells in the control condition showed no change in YFP/CFP ratio of the DORA RhoA biosensor. Stimulation of cells transfected with GEFT also showed a similar response on the DORA RhoA biosensor (Supplemental Fig. 2A), which was completely inhibited if cells were incubated with the specific Gαq inhibitor FR900359^35^ (Supplemental Fig. 2B). This is in line with earlier results obtained with p63RhoGEF^580^ 21. Hardly any change in YFP/CFP ratio of the DORA Rac biosensor was observed after stimulation of cells transfected with either p63RhoGEF^580^ or p63RhoGEF^619^ compared to control cells transfected only a cytosolic RFP (Fig. 3B). Interestingly, we found that cells transfected with either p63RhoGEF^580^ or p63RhoGEF^619^ showed a robust reversible increase in YFP/CFP ratio of the DORA Cdc42 biosensor after stimulation with histamine and mepyramine. Control cells expressing the DORA Cdc42 sensor and a cytosolic RFP did not show any change in YFP/CFP ratio upon stimulation with histamine and mepyramine (Fig. 3C). Overnight incubation with *Pertussis Toxin* (PTX)^36^ to inhibit Gαi activity did not change the response in cells transfected with the DORA Cdc42 biosensor and p63RhoGEF^619^, excluding a Gαi mediated effect (Supplemental Fig. 3A). Incubation of cells transfected with the DORA Cdc42 biosensor and p63RhoGEF^619^ with Gαq inhibitor FR900359 abrogated the histamine mediated response on the DORA Cdc42 biosensor, showing that this effect is mediated by the activation of p63RhoGEF^619^ via Gαq (Supplemental Fig. 3B).

Hence, despite the difference in their subcellular locations, p63RhoGEF^580^ and p63RhoGEF^619^ show surprisingly similar activation profiles on the Rho GTPase they can exert their GEF activity on.

### p63RhoGEF^619^ is recruited to the plasma membrane after activation of a GPCR

**Figure 4:**
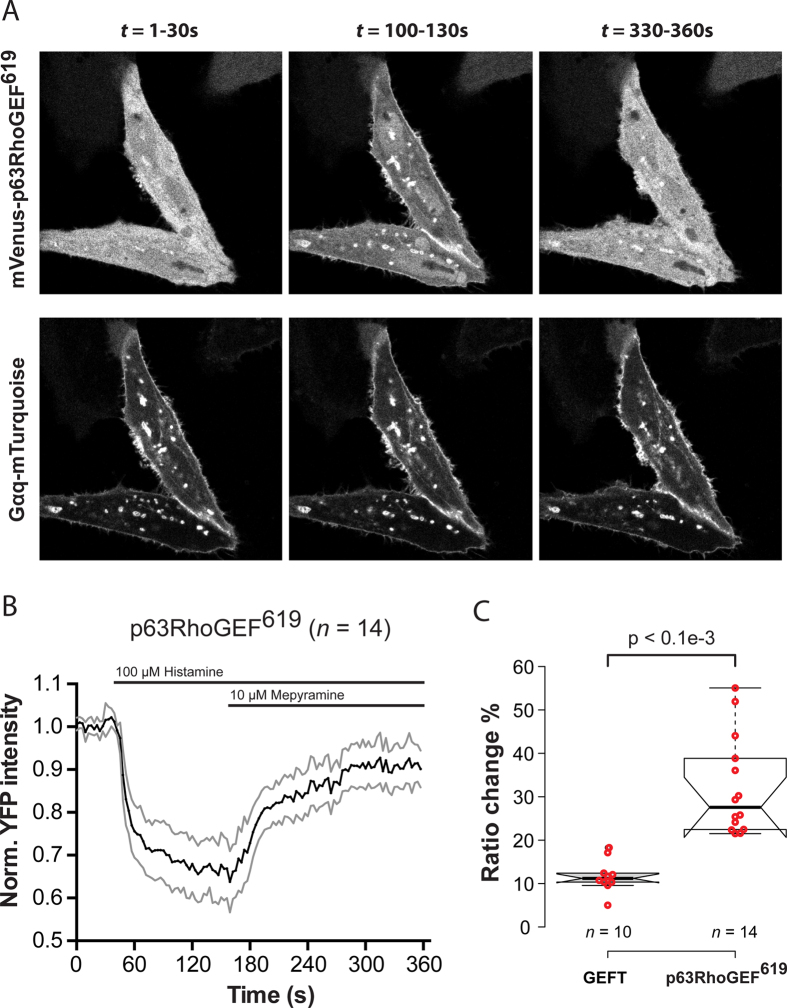
GPCR-Gαq mediated recruitment of p63RhoGEF^619^ to the plasma membrane. (**a**) Representative images at three time intervals of p63RhoGEF^619^-YFP (*top panels*) Relocation to the plasma membrane in HeLa cells transfected with p63RhoGEF^619^-YFP, Gαq-CFP and H_1_R-RFP (*bottom panels*; Gαq-CFP is shown for reference). (**b**) Quantification of the experiment shown in (**a**), trace shows the average normalized cytoplasmic YFP fluorescence over time (faded *grey* traces depict +/−95% CI). Cells were stimulated with histamine (100 μM) at t = 42 s and the response was antagonized by the addition of mepyramine (10 μM) at t = 162 s. (c) The median amplitude change of the YFP fluorescence at *t* = 120 seconds is quantified for YFP-GEFT and p63RhoGEF^619^-YFP. Centerlines of the boxplot in (**c**) represent the median values and notches represent the 95% confidence interval for each median. The p-values were determined by a two-tailed Mann-Whitney test. Width of the individual images in (**a**) is 86 μm.

Since we measured similar Rho GTPase activation profiles with a cytosolic (p63RhoGEF^619^) and plasma membrane (p63RhoGEF^580^) located variant of the same GEF, we set out to explore the mechanism behind this unexpected observation in more detail. It is well known that LARG, PDZ-RhoGEF and p115RhoGEF, RhoGEFs that are activated via direct interaction with Gα_12/13_^37^, relocate to the plasma membrane upon activation^38^. To investigate whether p63RhoGEF^619^ also relocates to the plasma membrane upon activation of Gαq, we used real-time confocal microscopy to inspect the subcellular location p63RhoGEF^619^ during stimulation of endogenous histamine-1-receptors. Pilot experiments indicated that the ectopic expression of p63RhoGEF^619^ leads to a quick saturation of available binding sites of endogenously activated Gαq. To overcome this, we transfected HeLa cells with p63RhoGEF^619^-YFP, Gαq-CFP and H_1_R-RFP. After stimulation of the cells with histamine we observed a rapid relocation of p63RhoGEF^619^ to the plasma membrane, which was reversed upon addition of mepyramine (Fig. 4A). This response was quantified by drawing ROIs in the cytoplasm of individual cells (Fig. 4B). Upon stimulation of cells transfected with YFP-GEFT, Gαq-CFP and H_1_R-RFP we observed a similar reversible relocation of GEFT to the plasma membrane, albeit with a lower amplitude (Fig. 4C, Supplemental Fig. 4A). We observed similar relocation kinetics for p63RhoGEF^619^ and GEFT when cells were co-transfected with both constructs at the same time (RFP-GEFT, p63RhoGEF^619^-YFP, Gαq-CFP and H_1_R-untagged) (Supplemental Fig. 4B). From this data we conclude that the ARHGEF25 isoform p63RhoGEF^619^ and splice variant GEFT, which reside in the cytosol in unstimulated cells, relocate to the PM upon activation of Gαq mediated signaling.

### Full activation of RhoGEF activity requires heterotrimeric G-protein signaling

**Figure 5:**
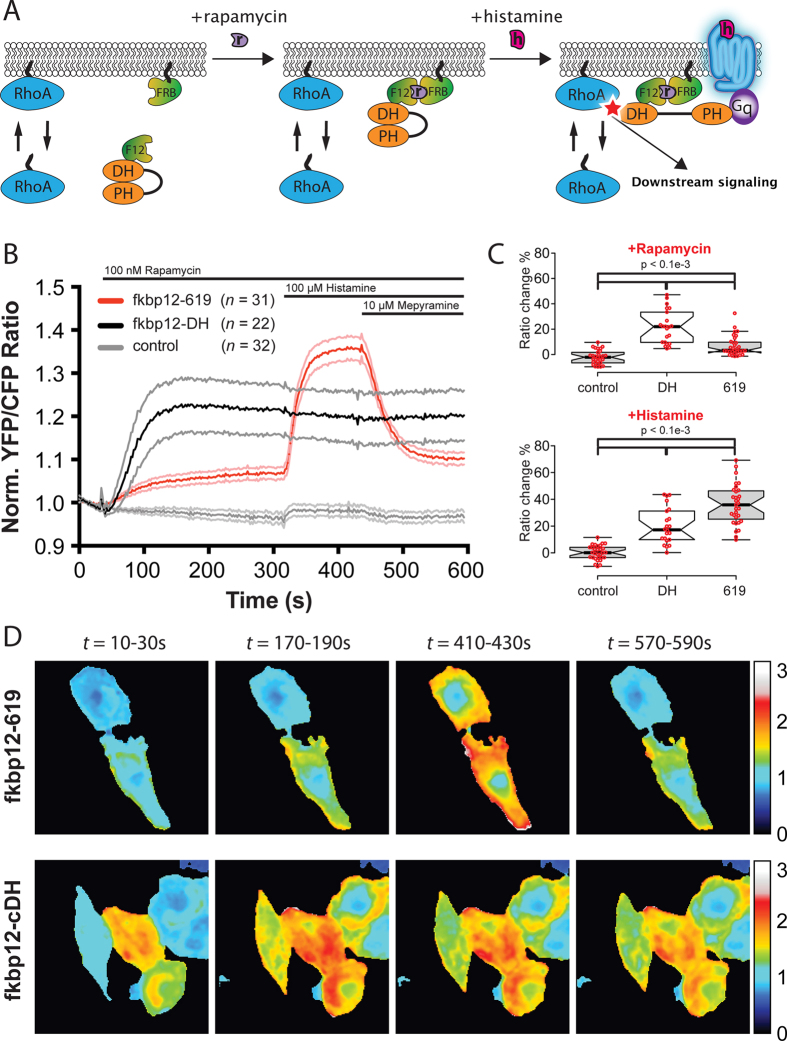
Activity of p63RhoGEF^619^ towards RhoA, separating activation by recruitment to the plasma membrane from allosteric activation. (**a**) Schematic overview of the experiment. In the basal state, p63RhoGEF^619^-fkbp12 is localized to the cytosol and its PH domain is folded onto the catalytic DH domain (*left*) (only the DH-PH domains of p63RhoGEF^619^ are shown in the cartoon to improve clarity). Upon stimulation with rapamycin, fkbp12 dimerizes with frb, causing a relocation of p63RhoGEF^619^-fkbp12 to the plasma membrane (*middle*). After stimulation with histamine, and subsequent activation of Gαq, the PH domain is released from the DH domain and p63RhoGEF^619^-fkbp12 is fully activated, leading to the activation of RhoA. (**b**) Average FRET measurements of HeLa cells transfected with the DORA RhoA-biosensor, Lck-frb-CFP(w66a) and p63RhoGEF^619^-RFP-fkbp12 (*n* = 31, *red*), RFP-fkbp12-cDH (*n* = 22, *black*) or RFP-fkbp12 transfected control cells (*n* = 32, *grey*). Cells were stimulated with rapamycin at *t* = 32 s, histamine at *t* = 312 s and mepyramine at *t* = 432 s. Time traces show the average ratio change of YFP/CFP fluorescence, normalized to baseline values (faded *grey* and *pink* traces depict +/−95% CI). (**c**) The median amplitude of the FRET ratio change in (**b**) after rapamycin addition (*t* = 300) (*top*) and histamine addition (*t *= 420 seconds) (*bottom*). (**d**) Examples of the experiment performed in (**b**) for the p63RhoGEF^619^-RFP-fkbp12 (*top*) and RFP-fkbp12-cDH (*bottom*) condition. YFP/CFP FRET ratio images are shown of the RhoA-biosensor for four different time intervals (10–30 s, 170–190 s, 410–430 s, 570–590 s). Center lines of the boxplot in (**c**) represent the median values and notches represent the 95% confidence interval for each median. The p-values were determined by a two-tailed Mann-Whitney test. Width of the individual images in (**d**) is 115 μm.

The observation that p63RhoGEF^619^ relocates to the plasma membrane argues for a dual role of activated Gαq where it both recruits p63RhoGEF^619^ to the plasma membrane and relieves it from its allosteric autoinhibition by the PH domain. To separate these events we made use of the rapamycin heterodimerization system^39^. In this approach, addition of rapamycin will recruit p63RhoGEF^619^ to the plasma membrane without allosteric activation by Gαq. Subsequent activation of a GPCR will result in allosteric activation without a contribution of relocation. By simultaneously monitoring the RhoGEF activity with the DORA RhoA biosensor we can quantify the contribution of both events to RhoGEF activity. To this end, HeLa cells transfected with the DORA RhoA biosensor, Lck-FRB-ECFP(W66A) and p63RhoGEF^619^-fkbp12-RFP were sequentially treated with rapamycin, histamine and mepyramine (Fig. 5A). After adding rapamycin, a gradual increase in YFP/CFP ratio of the RhoA biosensor is observed, most likely due to some constitutive activity of p63RhoGEF^619^ at the plasma membrane towards RhoA. (Fig. 5B,C red trace). Subsequent stimulation with histamine fully activates Gαq, leading to the allosteric release of the inhibitory PH domain of p63RhoGEF^619^, and rapid and large change in YFP/CFP ratio on the RhoA biosensor. The histamine-mediated activation of RhoA is then fully reversed upon addition of mepyramine (Fig. 5B, red trace, Fig. 5D). A similar experiment with the splice variant GEFT (RFP-fkbp12-GEFT) revealed an identical pattern in YFP/CFP ratio changes on the RhoA biosensor (Supplemental Fig. 5A). In contrast, when we recruited the isolated catalytic domain of ARHGEF25 (cDH-fkbp12-RFP) with rapamycin, we observed a large and fast change in ratio of YFP/CFP of the RhoA biosensor (Fig. 5B,C, black trace, Fig. 5D). The ratio did not change further upon stimulation with histamine or mepyramine, indicating that relocation of the isolated catalytic GEF domain of ARHGEF25 to the plasma membrane is sufficient for full activation of RhoA as previously reported^21^. Recruitment of an fkbp12-RFP serves as a control since this unit has no RhoGEF activity. Indeed, recruitment of fkbp12-RFP does not lead to any change in YFP/CFP ratio of the RhoA biosensor, whereas addition of histamine leads to a small reversible change in FRET ratio that can most likely be explained by the activation of the endogenous Gαq mediated GEF Trio^40^ (Fig. 5B, grey trace, Fig. 5C). Of note, when we compared the raw ratios of the RhoA biosensor in all conditions, we observed only an increased basal YFP/CFP ratio of the RhoA biosensor in cells transfected with cDH (Supplemental Fig. 5B). This increased basal ratio has been observed before by us^21^, and is likely due to the random interactions of the constitutive active cDH construct and the RhoA biosensor in the cytoplasm. The raw FRET ratio data also shows that the maximum YFP/CFP ratio change induced by rapamycin in the cDH condition is very comparable (25%) to the ratio change induced by rapamycin + histamine in the p63RhoGEF^619^/GEFT conditions (Supplemental Fig. 5B).

Together, from these results we conclude that recruitment of p63RhoGEF^619^ increases RhoGEF activity but that maximal stimulation requires allosteric activation via heterotrimeric G-protein signaling.

## Discussion

The two undisputed gene products of ARHGEF25, p63RhoGEF^580^ and p63RhoGEF^619^, display obvious differences in subcellular localization when expressed in unstimulated HeLa cells. Five putative palmitoylation sites at the N-terminus of p63RhoGEF^580^ target this isoform to the plasma membrane^22^,^23^, while p63RhoGEF^619^ resides in the cytoplasm. Interestingly, despite this difference in localization, we observed no difference in basal GEF activity towards RhoA. Surprisingly, both isoforms reversibly activate RhoA with similar magnitudes upon stimulation of Gαq coupled GPCRs. Closer inspection of p63RhoGEF^619^ revealed that this isoform dynamically relocates to the plasma membrane during GPCR stimulation on a seconds timescale. Of note, the RhoGEF Trio, which has a DH/PH domain homologous to p63RhoGEF, has been shown to relocate to the plasma membrane after activation of Gαq at much slower timescales^41^. These observations resemble the well-known relocation mechanism of the Gα_12/13_ mediated RhoGEFs p115RhoGEF, PDZ-RhoGEF and LARG^38^.

It was previously shown that recruitment of the isolated catalytic DH domain of p63RhoGEF from the cytosol to the plasma membrane leads to robust activation of RhoA^21^. By synthetically recruiting p63RhoGEF^619^ to the plasma membrane we could separate the effects of plasma membrane recruitment of p63RhoGEF^619^ on the one hand, and allosteric activation by Gαq on the other hand. P63RhoGEF^619^ mediated GEF activity towards RhoA is slightly enhanced when it is artificially recruited to the plasma membrane, but full GEF activity requires stimulation by Gαq. This reveals a double role for Gαq in cells, where it both recruits and allosterically activates P63RhoGEF^619^.

Interestingly, we also found GEF activity towards the Cdc42 of both p63RhoGEF isoforms, while we did not find evidence of Rac activation. Although Cdc42 and Rac activity of both p63RhoGEF^580^ 42 and p63RhoGEF^619^ 24 has been described before, these results are controversial^12^,^20^. Our results with the Gaq inhibitor FR900359 indicate that the Cdc42 activity most likely originates, directly or indirectly, from Gαq mediated activity.

The ARHGEF25 gene and its protein products are only found in the vertebrate subphylum. It seems likely that ARHGEF25 gene arose from a split gene event of either Trio^40^ or Kalirin^43^, which are both found in vertebrate predecessors, and contain a DH/PH cassette that is highly homologous in sequence to ARHGEF25. Interestingly, recent efforts with proteomics approaches^25^,^26^ have found expression of both p63RhoGEF^619^ and p63RhoGEF^580^ specifically in human tissues that also contain smooth muscle, including the brain (vasculature), fetal heart, uterus, reproductive tract, urinary tract, gastrointestinal tract and the retina. The fact that smooth muscle tissue has evolved only in vertebrates^44^, and p63RhoGEF function has been extensively linked to vascular smooth muscle cell function^16^,^17^,^28^,^45^ and hypertension^18^,^19^,^46^,^47^, provides strong evidence for co-evolution and a functional connection between p63RhoGEF and smooth muscle tissue. Which of the ARHGEF25 isoforms is expressed in vascular smooth muscle tissue, whether they can be specifically targeted and if one of isoforms is main cause of hypertensive pathogenicity, should be subject of future studies.

In conclusion, we compared subcellular localization and functional output of two isoforms of the gene ARHGEF25, p63RhoGEF^580^ and p63RhoGEF^619^. Interestingly, while the two isoforms display remarkable differences in subcellular localization, p63RhoGEF^580^ is located at the plasma membrane, and p63RhoGEF^619^ located in the cytosol, they activate the RhoGTPases RhoA and Cdc42 to a similar extend upon Gαq mediated GPCR stimulation. We found that p63RhoGEF^619^ relocates to the plasma membrane upon activation of Gαq, very similar to the mechanisms of Gα_12/13_ mediated RhoGEFs. Synthetic recruitment of p63RhoGEF^619^ to the plasma membrane slightly increases its GEF activity towards RhoA, but full activation requires allosteric activation by Gαq. The resemblance of signaling output between a cytoplasmic and membrane located GEF is striking. Future studies may reveal whether signaling output of the two variants is similar under physiological conditions.

## Methods

### Construction of fluorescent protein fusions

The CFP, RFP and YFP variants used in this study are mTurquoise, mCherry and mVenus, respectively. P63RhoGEF^580^-RFP, p63RhoGEF^580^-YFP, YFP-p63RhoGEF^580^, YFP-GEFT, RFP-GEFT, RFP- fkbp12, RFP- fkbp12-GEFT and RFP-p63RhoGEF^580^, RFP- fkbp12-cDH were constructed previously^21^,^22^. Gαq-mTurquoise and H_1_R-untagged^48^ were previously described. H_1_R-mCherry is described elsewhere^34^.

A membrane targeting sequence (derived from amino acid residue 1–10 of Lck; MGCVCSSNPE) was constructed by annealing^49^ two oligonucleotide linkers, 5′-ctagccaccatgggctgcgtgtgcagcagcaaccccgagcta-3′ and 5′-ccggtagctcggggttgctgctgcacacgcagcccatggtgg-3′, with sticky overhangs and inserting it into an mVenus-C1 plasmid cut with NheI and AgeI, resulting in Lck-mVenus. Lck-mTurquoise2 was obtained by exchanging mVenus for mTurquoise2 in the Lck-mVenus plasmid by cutting with AgeI and BsrGI.

In order to construct p63RhoGEF^619^-RFP, a plasmid containing the following nucleotide sequence was ordered (Eurofins): 5′-ggactcagatctcgagctc**aagctt**cgaattctgcagtcga cggtaccaccatgaagcccccggaccgc cccgcccctggccgcac tgaccggatactgggggtcatggggggcatgctgcgcgcatgcgccctccctgggcaggaggggcccccaaggagaagccctctagggttggtgggtaccgagccagagtctgaacgtacggagggagatcacagaagggatcgcgaacatgaggtcctcgccggggctctgcagcccga atcctattccattgcgggcagtgaggggagtatatcggcttctgctgcctccggtctggctgccccctctggccccagctctggcctcagctctggcccctgttccccaggccc cccagggcccgtcagtggcctgaggagatg gttggatcattccaaacattgtctcagtgtgg aaactgaggca**gacagtggtc**aggcagga-3′. Cutting the plasmid with HindIII and PshAI (restriction sites in bold and underlined) and ligating it into a plasmid containing p63RhoGEF^580^-RFP, cut with the same enzymes, resulted in p63RhoGEF^619^-RFP. P63RhoGEF^619^-YFP was obtained by swapping the RFP for YFP with AgeI and BsrGI. p63RhoGEF^619^-fkbp12-RFP was obtained by cutting p63RhoGEF^619^-RFP with HindIII and BsrGI and ligating fkbp12, cut with the same enzymes from N1-fkbp12-mTurquoise2, in between the sequence for p63RhoGEF^619^ and RFP.

The Lck-FRB-ECFP(W66A) was a kind gift from M. Putyrski^39^. The DORA Rho GTPase sensors were described previously; DORA-RhoA^21^,^50^, DORA-Rac1 sensor^32^ and the DORA-Cdc42 sensor^33^. N1-fkbp12-mTurquoise2 was a kind gift from Kevin Batenburg. Plasmids constructed in this study will be made available through Addgene: https://www.addgene.org/Dorus_Gadella/

### Cell Culture & Sample Preparation

HeLa cells (American Tissue Culture Collection: Manassas, VA, USA) were cultured using Dulbecco’s Modified Eagle Medium (DMEM) supplied with Glutamax, 10% FBS, Penicillin (100 U/ml) and Streptomycin (100 μg/ml). Cell culture, transfection and live cell microscopy conditions were performed as previously described^21^.

### Widefield microscopy

Ratiometric FRET measurements were performed using a previously described wide-field fluorescence microscope^21^. Typical exposure times ranged from 50 ms to 150 ms, and camera binning was set to 4 × 4. Fluorophores were excited with 420 nm light (slit width 30 nm) and reflected onto the sample by a 455DCLP dichroic mirror and CFP emission was detected with a BP470/30 filter, and YFP emission was detected with a BP535/30 filter by rotating the filter wheel. All acquisitions were corrected for background signal and bleedthrough of CFP emission in the YFP channel (around 55% of the intensity measured in the CFP channel).

In dynamic experiments, cells were stimulated with 100 μM histamine (Sigma-Aldrich) and 10 μM Mepyramine (Sigma-Aldrich) or 100 nM Rapamycin (LC Laboratories, Woburn, USA). Where indicated, cells were incubated with 100 ng/ml Pertussis Toxin (PTX) (Sigma Aldrich) overnight. The Gαq inhibitor FR900359^35^ was added to cells 2 hours before the measurements started at a concentration of 2 μM and was purchased from the University of Bonn ( http://www.pharmbio.uni-bonn.de/signaltransduktion).

### Confocal microscopy

Localization and relocation experiments were performed using a Nikon A1 confocal microscope equipped with a 60x oil immersion objective (Plan Apochromat VC, NA 1.4). The pinhole size was set to 1 Airy unit (<0.8 μm). 457 nm, 514 nm and 561 nm laser lines were reflected onto the sample by a 457/514/561 dichroic mirror. To avoid bleed-through, images were acquired with sequential line scanning modus. CFP, YFP and RFP emission was detected using BP482/35, BP540/30 and BP595/50 emission filters respectively.

### Data Analysis

ImageJ (National Institute of Health) was used to analyze the raw microscopy images. Further processing of the data was done in Excel (Microsoft Office) and graphs and statistics were conducted using Graphpad version 6.0 for Mac, GraphPad Software, La Jolla California USA, www.graphpad.com. The non-parametric two-tailed Mann-Whitney test was used to determine p values. We list exact p-values where possible, unless p values < 0.1e-3, since GraphPad does not give exact p-values in these situations.

Boxplots were generated online, using the website http://boxplot.tyerslab.com/, showing individual data points, the median (center line) and 95% confidence intervals (notches). Box limits indicate the 25^th^ and 75^th^ percentiles as determined by R software and whiskers extend 1.5 times the interquartile range (IQR) from the 25^th^ and 75^th^ percentiles. The notches are defined as +/−1.58 * IQR/ √(n) and represent the 95% confidence interval for each median.

The p-values and 95% confidence intervals are determined to aid the interpretation and are not used as thresholds to determine biological significance^51^.

To compare the intensities of the GEF, PM marker and cytosolic marker, line plots in Fig. 1 were made by normalizing the fluorescent intensities in the different channels to the maximum value.

### Sequence Analysis

Protein sequences from other species were obtained from -and evolutionary analysis of p63RhoGEF^580^ and p63RhoGEF^619^ was performed using a combination of BLAST (NCBI), Ensembl genome browser^52^ and the UCSC human genome browser^53^. Sequences were aligned and visualized using Jalview^54^.

## Additional Information

**How to cite this article**: van Unen, J. *et al*. Kinetics of recruitment and allosteric activation of ARHGEF25 isoforms by the heterotrimeric G-protein Gαq. *Sci. Rep.* **6**, 36825; doi: 10.1038/srep36825 (2016).

**Publisher’s note:** Springer Nature remains neutral with regard to jurisdictional claims in published maps and institutional affiliations.

## Supplementary Material

Supplementary Information

Supplementary Movie 1

Supplementary Movie 2
