# Supplementary material for: Kinetics of recruitment and allosteric activation of ARHGEF25 isoforms by the heterotrimeric G-protein Gαq
Source: Sci Rep. 2016 Nov 11;6:36825. doi: 10.1038/srep36825 (PMC5105084; doi:10.1038/srep36825)
Supplement: Supplementary Information [file srep36825-s1.pdf]

Supplementary Information

Kinetics of recruitment and allosteric activation of ARHGEF25 isoforms by the heterotrimeric G-protein Gαq

Jakobus van Unen, Taofei Yin, Yi I. Wu, Marieke Mastop, Theodorus W.J. Gadella Jr., Joachim Goedhart

Supplementary Figure 1

```
Human p63RhoGEF  (619aa)    1  MKPPDRPAPGRTDRILGVMGGMLRACALPGQEGPPRRSPLGLVGTEPESERTEGDHRRDREHEVLAGALQPESYSIAGS   79
Human p63RhoGEF  (580aa)    1  ---------------------------------------MRGGHKGGRCACPRVIRKVLAKCGCCFARGGRESYSIAGS   40
Human GEFT       (474aa)       -------------------------------------------------------------------------------

Human p63RhoGEF  (619aa)   80  EGSISASAASGLAAPSGPSSGLSSGPCSPGPPGPVSGLRRWLDHSKHCLSVETEADSGQAGPYENWMLEPALATGEELP  158
Human p63RhoGEF  (580aa)   41  EGSISASAASGLAAPSGPSSGLSSGPCSPGPPGPVSGLRRWLDHSKHCLSVETEADSGQAGPYENWMLEPALATGEELP  119
Human GEFT       (474aa)    1  ------------------------------------------------------------------MLEPALATGEELP   13

Human p63RhoGEF  (619aa)  159  ELTLLTTLLEGPGDKTQPPEEETLSQAPESEEEQKKKALERSMYVLSELVETEKMYVDDLGQIVEGYMATMAAQGVPES  237
Human p63RhoGEF  (580aa)  120  ELTLLTTLLEGPGDKTQPPEEETLSQAPESEEEQKKKALERSMYVLSELVETEKMYVDDLGQIVEGYMATMAAQGVPES  198
Human GEFT       (474aa)   14  ELTLLTTLLEGPGDKTQPPEEETLSQAPESEEEQKKKALERSMYVLSELVETEKMYVDDLGQIVEGYMATMAAQGVPES   92

Human p63RhoGEF  (619aa)  238  LRGRDRIVFGNIQQIYEWHRDYFLQELQRCLKDPDWLAQLFIKHERRLHMYVVYCQNKPKSEHVVSEFGDSYFEELRQQ  316
Human p63RhoGEF  (580aa)  199  LRGRDRIVFGNIQQIYEWHRDYFLQELQRCLKDPDWLAQLFIKHERRLHMYVVYCQNKPKSEHVVSEFGDSYFEELRQQ  277
Human GEFT       (474aa)   93  LRGRDRIVFGNIQQIYEWHRDYFLQELQRCLKDPDWLAQLFIKHERRLHMYVVYCQNKPKSEHVVSEFGDSYFEELRQQ  171

Human p63RhoGEF  (619aa)  317  LGHRLQLNDLLIKPVQRIMKYQLLLKDFLKYYNRAGMDTADLEQAVEVMCFVPKRCNDMMTLGRLRGFEGKLTAQGKLL  395
Human p63RhoGEF  (580aa)  278  LGHRLQLNDLLIKPVQRIMKYQLLLKDFLKYYNRAGMDTADLEQAVEVMCFVPKRCNDMMTLGRLRGFEGKLTAQGKLL  356
Human GEFT       (474aa)  172  LGHRLQLNDLLIKPVQRIMKYQLLLKDFLKYYNRAGMDTADLEQAVEVMCFVPKRCNDMMTLGRLRGFEGKLTAQGKLL  250

Human p63RhoGEF  (619aa)  396  GQDTFWTEPEAGGLLSSRGRERRVFLFEQIIIFSEALGGGVRGGTQPGYVYKNSIKVSCLGLEGNLQGDPCRFALTSR  474
Human p63RhoGEF  (580aa)  357  GQDTFWTEPEAGGLLSSRGRERRVFLFEQIIIFSEALGGGVRGGTQPGYVYKNSIKVSCLGLEGNLQGDPCRFALTSR  435
Human GEFT       (474aa)  251  GQDTFWTEPEAGGLLSSRGRERRVFLFEQIIIFSEALGGGVRGGTQPGYVYKNSIKVSCLGLEGNLQGDPCRFALTSR  329

Human p63RhoGEF  (619aa)  475  GPEGGIQRYVLQAADPAISQAWIKHVAQILESQRDFLNALQSPIEYQRRESQTNSLGRPRGPGVGSPGRIRLGDQAQGS  553
Human p63RhoGEF  (580aa)  436  GPEGGIQRYVLQAADPAISQAWIKHVAQILESQRDFLNALQSPIEYQRRESQTNSLGRPRGPGVGSPGRIRLGDQAQGS  514
Human GEFT       (474aa)  330  GPEGGIQRYVLQAADPAISQAWIKHVAQILESQRDFLNALQSPIEYQRRESQTNSLGRPRGPGVGSPGRIRLGDQAQGS  408

Human p63RhoGEF  (619aa)  554  THTPINGSLPSLLLSPKGEVARALLPLDKQALGDIPQAPHDSPPVSPTPKTPPCQARLAKLDEDEL               619
Human p63RhoGEF  (580aa)  515  THTPINGSLPSLLLSPKGEVARALLPLDKQALGDIPQAPHDSPPVSPTPKTPPCQARLAKLDEDEL               580
Human GEFT       (474aa)  409  THTPINGSLPSLLLSPKGEVARALLPLDKQALGDIPQAPHDSPPVSPTPKTPPCQARLAKLDEDEL               474
```

Full protein sequence alignment of all three ARHGEF25 isoforms. A green and purple box mark the unique translated exons from p63RhoGEF619. The unique translated exon from p63RhoGEF^{580} is marked by a red box. The common exon between p63RhoGEF^{619} and p63RhoGEF^{580} is marked by a blue box. The remaining sequence is identical for all three isoforms.

Supplementary Figure 2

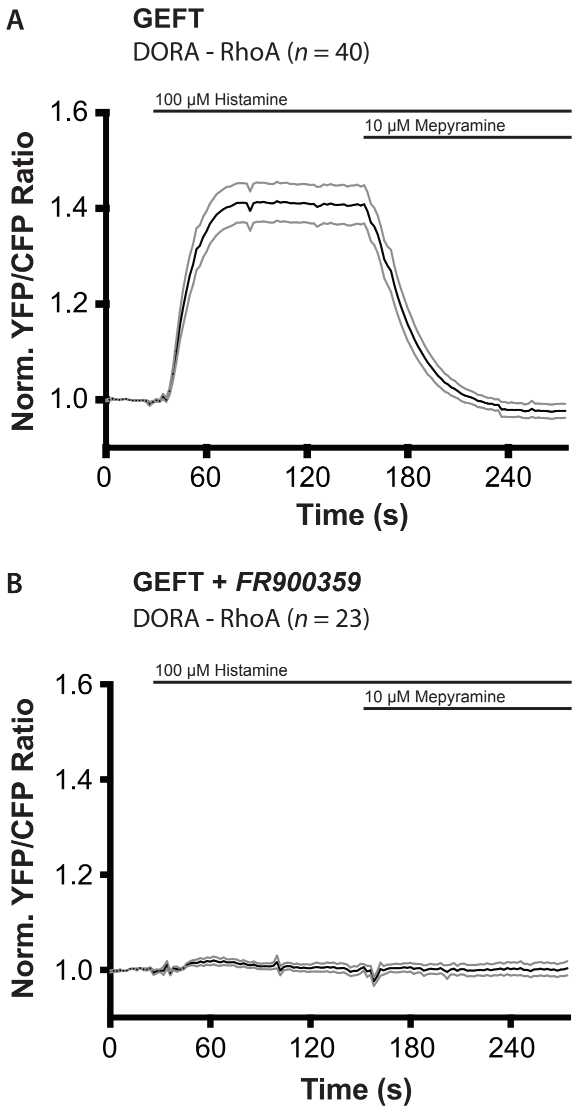

(A) Average FRET ratio time traces of HeLa cells transfected with the DORA RhoA-biosensor and RFP-GEFT (*n* = 40) show a fast reversible increase in YFP/CFP ratio, indicating rapid GTP loading of RhoA upon GPCR stimulation.
(B) Control cells transfected with the DORA RhoA-biosensor and RFP-GEFT (*n* = 23) that were incubated for 2 hours with 2μM FR900959 do not show any change in YFP/CFP ratio. Cells were stimulated with histamine (100μM) at t = 32s and the response was antagonized by the addition of mepyramine (10μM) at t = 152s. Time traces show the average ratio change of YFP/CFP fluorescence, normalized to baseline values (faded *grey* traces depict +/- 95% CI).

Supplementary Figure 3

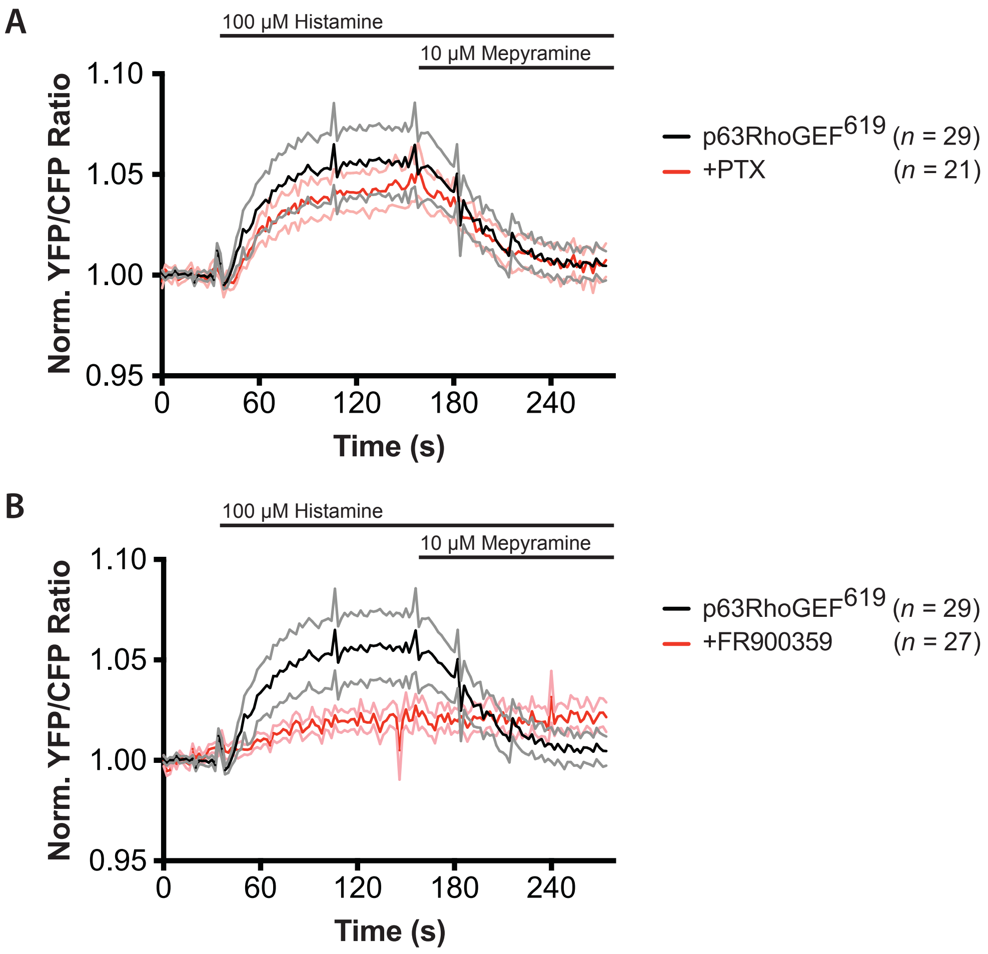

(A) Cells transfected with the DORA Cdc42-biosensor and RFP-p63RhoGEF^{619} (*n* = 21) that were incubated overnight with 100ng/ml PTX show reversible change in YFP/CFP ratio that is comparable to the untreated condition (the trace of RFP-p63RhoGEF^{619} (*n* = 29) from figure 3 is shown in *black*).
(B) Cells transfected with the DORA Cdc42-biosensor and RFP-p63RhoGEF^{619} (*n* = 27) that were incubated for 2 hours with 2µM FR900959 show a delayed and irreversible change in YFP/CFP ratio (the trace of RFP-p63RhoGEF^{619} (*n* = 29) from figure 3 is shown in *black*). Cells were stimulated with histamine (100µM) at t = 32s and the response was antagonized by the addition of mepyramine (10µM) at t = 152s. Time traces show the average ratio change of YFP/CFP fluorescence, normalized to baseline values (faded *grey* and *pink* traces depict +/- 95% CI).

Supplementary Figure 4

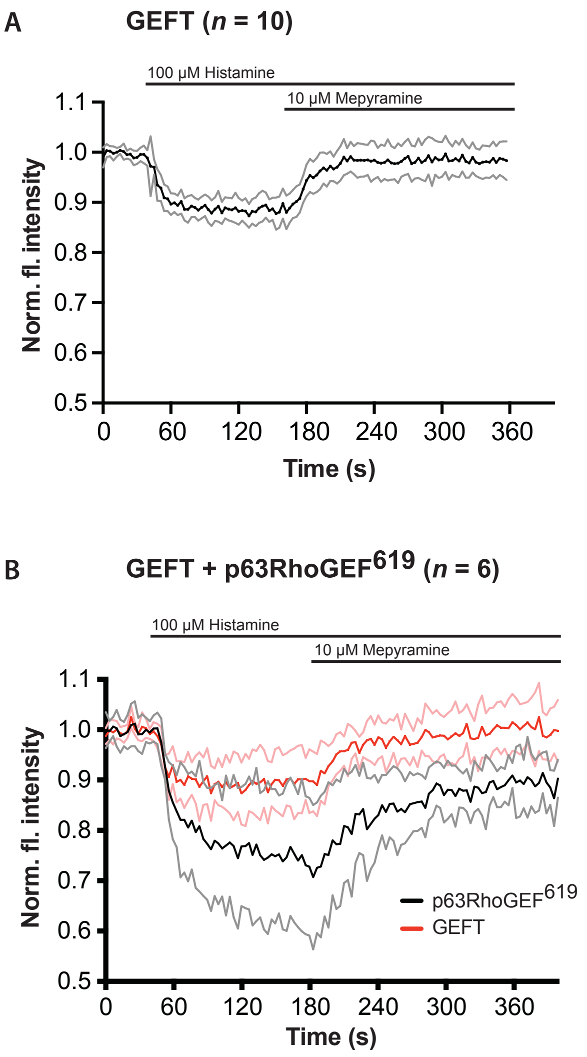

(A) Time trace of HeLa cells transfected with GEFT-YFP, Gαq-CFP and H_1R-RFP shows the average normalized YFP fluorescence over time (faded *grey* traces depict +/- 95% CI). Cells were stimulated with histamine (100μM) at t = 42s and the response was antagonized by the addition of mepyramine (10μM) at t = 162s. (B) Time trace of HeLa cells transfected with GEFT-RFP, YFP-p63RhoGEF^{619}, Gαq-CFP and H_1R-* shows the average normalized YFP and RFP fluorescence over time (faded *grey* and *pink* traces depict +/- 95% CI). Cells were stimulated with histamine (100μM) at t = 42s and the response was antagonized by the addition of mepyramine (10μM) at t = 182s.

Supplementary Figure 5

A

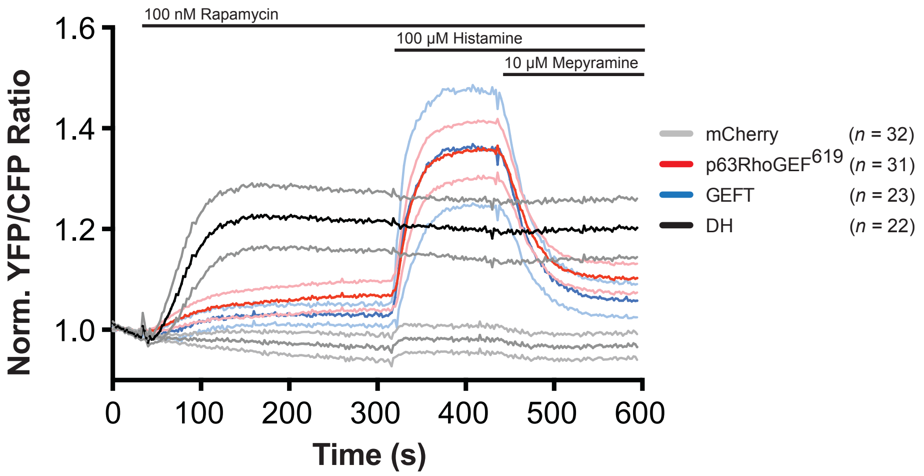

B

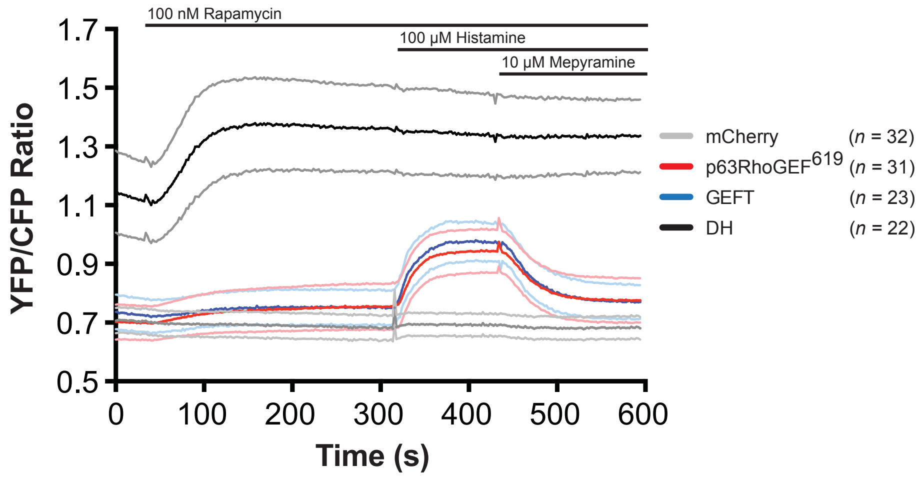

(A) Average FRET measurements of HeLa cells transfected with the DORA RhoA-biosensor, Lck-frb-CFP(w66a) and GEFT-RFP (*n* = 23, *blue*). Cells were stimulated with rapamycin at *t* = 32s, histamine at *t* = 312s and mepyramine at *t* = 432s. For reference, the RFP-fkbp12-p63RhoGEF^{619} (*n* = 31, *red*), RFP-fkbp12-cDH (*n* = 22, *black*) and RFP-fkbp12 transfected control cells (*n* = 32, *grey*) from figure 5 are also shown in their respective colors. Cells were stimulated with rapamycin at *t* = 32s, histamine at *t* = 312s and mepyramine at *t* = 432s. Time traces show the average ratio change of YFP/CFP fluorescence, normalized to baseline values (faded *grey, blue* and *pink* traces depict +/- 95% CI). (B) The same traces as in (A), but not normalized to their respective baseline ratios.

Supplementary Movies

Supplementary Video 1: Histamine induced relocation of $p63RhoGEF^{619}$ to the plasma membrane. HeLa cells transfected with YFP-$p63RhoGEF^{619}$, Gαq-CFP and H_1R-RFP were stimulated with histamine (100µM) at t = 42s and the response was antagonized by the addition of mepyramine (10µM) at t = 162s.
Movie shows the YFP fluorescence over time.

Supplementary Video 2: Histamine induced relocation of GEFT to the plasma membrane. HeLa cells transfected with GEFT-YFP, Gαq-CFP and H_1R-RFP were stimulated with histamine (100µM) at t = 56s and the response was antagonized by the addition of mepyramine (10µM) at t = 192s. Movie shows the YFP fluorescence over time.
